# Increased hypolipidemic benefits of *cis*-9, *trans*-11 conjugated linoleic acid in combination with *trans*-11 vaccenic acid in a rodent model of the metabolic syndrome, the JCR:LA*-cp *rat

**DOI:** 10.1186/1743-7075-7-60

**Published:** 2010-07-16

**Authors:** M Miriam Jacome-Sosa, Jing Lu, Ye Wang, Megan R Ruth, David C Wright, Martin J Reaney, Jianheng Shen, Catherine J Field, Donna F Vine, Spencer D Proctor

**Affiliations:** 1 Metabolic and Cardiovascular Diseases Laboratory, University of Alberta, Edmonton, AB, T6G 2P5, Canada; 2 Alberta Institute for Human Nutrition, University of Alberta, Edmonton, AB, T6G 2P5, Canada; 3 Alberta Diabetes Institute, University of Alberta, Edmonton, AB, T6G 2P5, Canada; 4 Department of Applied Microbiology and Food Science, University of Saskatchewan, Saskatoon, SK, S7N 5A8, Canada

## Abstract

**Background:**

Conjugated linoleic acid (*cis*-9, *trans*-11 CLA) and *trans*-11 vaccenic acid (VA) are found naturally in ruminant-derived foods. CLA has been shown to have numerous potential health related effects and has been extensively investigated. More recently, we have shown that VA has lipid-lowering properties associated with reduced hepatic lipidogenesis and chylomicron secretion in the JCR:LA*-cp *rat. The aim of this study was to evaluate potential additional hypolipidemic effects of purified forms of CLA and VA in an animal model of the metabolic syndrome (the JCR:LA-*cp *rat).

**Methods:**

Twenty four obese JCR:LA-*cp *rats were randomized and assigned to one of three nutritionally adequate iso-caloric diets containing 1% w/w cholesterol and 15% w/w fat for 16 wk: 1) control diet (CD), 2) 1.0% w/w *cis*-9, *trans*-11 CLA (CLA), 3) 1.0% w/w VA and 1% w/w *cis*-9, *trans*-11 CLA (VA+CLA). Lean rats were fed the CD to represent normolipidemic conditions.

**Results:**

Fasting plasma triglyceride (TG), total cholesterol and LDL-cholesterol concentrations were reduced in obese rats fed either the CLA diet or the VA+CLA diet as compared to the obese control group (p < 0.05, p < 0.001; p < 0.001, p < 0.01; p < 0.01, p < 0.001, respectively). The VA+CLA diet reduced plasma TG and LDL-cholesterol to the level of the normolipidemic lean rats and further decreased nonesterified fatty acids compared to the CLA diet alone. Interestingly, rats fed the VA+CLA diet had a higher food intake but lower body weight than the CLA fed group (P < 0.05). Liver weight and TG content were lower in rats fed either CLA (p < 0.05) or VA+CLA diets (p < 0.001) compared to obese control, consistent with a decreased relative protein abundance of hepatic acetyl-CoA carboxylase in both treatment groups (P < 0.01). The activity of citrate synthase was increased in liver and adipose tissue of rats fed, CLA and VA+CLA diets (p < 0.001) compared to obese control, suggesting increased mitochondrial fatty acid oxidative capacity.

**Conclusion:**

We demonstrate that the hypolipidemic effects of chronic *cis*-9, *trans*-11 CLA supplementation on circulating dyslipidemia and hepatic steatosis are enhanced by the addition of VA in the JCR:LA-*cp *rat.

## Introduction

Conjugated linoleic acid (CLA) is a term that refers to diverse positional and geometrical isomers of linoleic acid and its numerous health related effects have been extensively investigated. CLA was first described as a potent anti-carcinogenic component and more recently has been associated with improving dyslipidemia, insulin sensitivity and the pro-inflammatory state related to obesity and the metabolic syndrome [1,2]. However, some animal studies (specifically those using mouse models), in addition to a handful of clinical trials, have indicated that the major isomers found in CLA mixtures (*cis*-9, *trans*-11 CLA and *trans*-10, *cis*-12 CLA) are responsible for different physiological effects [1-10].

CLA is found naturally in ruminant-derived lipids and *cis*-9, *trans*-11 CLA is the major natural isoform, accounting for about 80-90% of the total CLA isomers [11]. *Trans*-11 vaccenic acid (VA) is the precursor to endogenous synthesis of the *cis*-9, *trans*-11 CLA isomer in rats [12,13] and humans [14], and is the predominant isomer of the total *trans *fatty acids found in ruminant-derived fats such as dairy and meat products. We have shown previously that unlike industrially produced *trans *fatty acids, VA has lipid-lowering properties associated with reduced hepatic lipogenesis and chylomicron secretion in the obese and insulin resistant JCR:LA-*cp *rat [15]. Interestingly, our observations indicate that VA has neutral effects under normolipidemic conditions and induces hypotriglyceridemic effects under conditions of dyslipidemia [16]. We also observed that VA supplementation for 16 weeks had a greater potential to influence lipoprotein metabolism [15] compared to a shorter term feeding [16]. These findings are supported by several clinical [17-19] and animal studies [20-24] showing that dietary *trans *fats derived from ruminants have either neutral or beneficial effects on cardiovascular disease risk factors compared to industrially produced *trans *fats.

As a result of the increasing evidence associating CLA and more recently VA with health benefits, there has been a growing interest to increase the concentrations of these natural *trans *fats in meat and dairy products [25-28]. Interestingly, both VA and CLA (*cis*-9, *trans*-11) can account for more than 15% of the total fat in naturally enhanced dairy products [26] which could provide an additional health value to animal-derived fats. Consequently, in this study we hypothesized that chronic supplementation with both CLA and VA would enhance the lipid lowering effects to improve whole body lipid metabolism. Therefore, the aim of this study was to evaluate the effect of dietary supplementation with purified forms of both *cis*-9, *trans*-11 CLA and VA on impaired lipid metabolism in an established animal model of the metabolic syndrome, the JCR:LA*-cp *rat.

## Materials and methods

### Animals and diets

All experimental procedures were approved by the University of Alberta Animal Ethics Committee and conducted in accordance with the Canadian Council on Animal Care. Twenty four male obese JCR:LA-*cp *rats (*cp/cp*) were raised in our established breeding colony at the University of Alberta as previously described [29]. At 3 wk of age, rats were transferred from the isolated breeding colony areas to an individually ventilated caging environment (TecniplastTM, Exton PA, USA) and had access to a standard rat chow diet (5001, PMI Nutrition International) (see Additional file 1). At 8 wk of age, rats (n = 8) were randomized and assigned to one of three diets for 16 wk (control and experimental diets) and had free access to food and water. Age and weight matched lean littermates (n = 8) were fed the control diet to mimic cholesterol/high fat feeding under normolipidemic conditions. Food intake and body weight (BW) were monitored weekly throughout the study. At 23 wk of age, a meal tolerance test (MTT) as previously described [30], was performed in four randomly chosen rats from control and treatment groups in order to determine plasma glucose and insulin concentrations after a meal. We also performed an oral fat challenge test (OFC), as previously described [29] in four additional rats from each group. At the end of the treatment period (24 wk of age), rats were fasted overnight and anesthetized using isofluorane anesthesia. Plasma was sampled from the left ventricle and heart, liver and fat pads were excised, weighted and immediately frozen at -80°C until analysis. Adipose fatty acid composition was measured from total triglyceride on the epidydimal fat pad as previously described [16].

Three iso-caloric diets were prepared with a constant polyunsaturated to saturated fatty acid ratio (P:S) of 0.4. A control diet (CD) was supplemented (w/w) with 1% cholesterol and contained 42% of energy from carbohydrate, 23.7% from protein and 34.3% from fat. Experimental diets were prepared by adjusting the lipid composition of the CD to provide 1.0% w/w of *cis*-9, *trans*-11 CLA alone (CLA), both 1% of VA and 1% w/w of *cis*-9, *trans*-11 CLA (VA+CLA). Semi-purified *cis*-9, *trans*-11 CLA (G-c9t11 80:20) containing 59.8% of *cis*-9, *trans*-11 CLA and 14.4% of *trans*-10, *cis*-12 CLA was kindly provided by Lipid Nutrition. The amount of CLA and VA (1% w/w) was chosen based on previous studies allowing for metabolic sufficiency while maintaining a normal dietary fatty acid proportion [15-17,31]. Purified VA was synthesized by a chemical alkali isomerisation from linoleic acid-rich vegetable oil [32]. The diet mixture was extruded into pellets, dried at RT°C and stored at 4°C. Fatty acid composition of the three diets was confirmed by gas chromatograph analysis [28] of the fat blend samples (Table 1).

**Table 1:** Fatty acid composition (% of total fatty acids) of control and experimental diets

Fatty acid	Control diet (CD)	CLA diet	VA+CLA diet
C16:0	9.1	8.5	9.1
C18:0	47.3	46.9	44.3
18:1 *t-*11 (VA)	ND	ND	5.6
18:1 *c-*9 (OA)	17.3	11.4	10.5
18:1 *c-*11	ND	0.5	0.5
C18:2 n6 (LA)	23.3	23.4	20.4
C18:3 n3 (ALA)	1.6	1.7	1.6
CLA *c-*9, *t-*11	0	5.2	3.9
CLA *t-*10, *c-*12	ND	1.1	0.8
other CLA	ND	0.3	0.3
Summary			
∑ total SFA^2^	57.2	56.3	54.2
∑ C12:0, C14:0, C16:0^3^	9.1	8.5	9.1
∑ *cis *MUFA^4^	17.4	12.0	11.1
∑ PUFA^5^	25.0	25.1	22.0
∑ n-6 PUFA	23.4	23.4	22.0
∑ n-3 PUFA	1.6	1.7	1.6
P/S ratio^6^	0.4	0.4	0.4
∑ CLA	0.0	6.6	5.0

^1. ^No detectable

^2 ^Sum of all saturated fatty acids

^3 ^Sum of lauric, myristic and palmitic acids

^4 ^*cis *MUFA, sum of all monounsaturated excepting *trans *fatty acids

^5 ^Sum of all polyunsaturated fatty acids excepting CLA

^6 ^Ratio of polyunsaturated to saturated fatty acids

### Plasma biochemical components

The concentration of biochemical parameters in fasting plasma from lean and obese groups were assessed using commercially available homogenous, enzymatic colorimetric assays. Triglyceride (TG) (Wako Pure Chemical Industries, catalog no. 998-40391, 0.01 mmol/L minimum), Total cholesterol (TC) (Wako Pure Chemical Industries, catalog no. 993-00404, 0.002 mmol/L minimum), LDL cholesterol (LDL-C) (Wako Pure Chemical Industries, catalog no. 993-00404, 0.03-10.4 mmol/L) and nonesterified fatty acids (NEFA) (HR Series NEFA-HR, catalog no. 999-34691, Wako Diagnostics) were measured using direct colorimetric chemical enzymatic reactions. Plasma glucose was measured as per the glucose oxidase method (Diagnostic Chemical, catalog no. 220-32, 0.03-33.3 mmol/L) and plasma insulin was determined using commercially available enzymatic immunoassays for rodents (Ultrasensitive rat insulin ELISA, Mercodia, catalog no. 80-INSRTU-E01, 0.03-1.0 pmol/L). Samples were analyzed in triplicate using assay kits from a single lot and performed in one single batch.

### Tissue homogenization and hepatic TG

Liver and adipose tissue samples (0.5 g) were homogenized in 200 μL lysis buffer [PBS (pH 7.4) with 1.5% TritonX-100 and 1% protease inhibitor cocktail (Sigma)] and and hepatic TG levels were determined by a commercially available enzymatic colorimetric assay (Wako Pure Chemical Industries, catalog no. 998-40391, 0.01 mmol/L minimum), using an aliquot of the whole homogenate and adjusting by the protein concentration of the homogenate [15]. The remainder of the homogenate was centrifuged at 700 g for 15 min and the supernatant was collected and stored at -80C for western blot and citrate synthase activity analysis.

### Hepatic and adipose tissue citrate synthase activity

Citrate synthase activity in liver and adipose tissue samples was determined using a commercially available kit from Sigma (catalog no. CS0720). The coefficient of variation of this assay in our laboratory is < 10%. The citrate synthase activity was expressed as μmol/min/g protein.

### Relative protein abundance of lipogenic enzymes

Hepatic acetyl-CoA carboxylase-1 (ACC-1) and fatty acid synthase (FAS) were determined by western blot analysis as described elsewhere [33] with few modifications [15]. ACC-1 and FAS relative abundance were normalized based on the respective β-actin protein mass (internal control).

### Statistical analysis

Statistical analysis was performed using the Graph pad Prism software, version 4.0. Data was tested for normal distribution and one-way ANOVA followed by Tukey post-hoc tests were used to identify differences among both lean and obese controls and treatment groups (CLA and VA+CLA). Post-prandial glucose and insulin metabolism as well as post-prandial TG response were assessed by area under the curve (AUC) analysis. Fasting concentrations of these parameters were further subtracted from the total AUC to yield the incremental area under the curve (iAUC). Results are expressed as means ± SEM and the level of significance was set at p < 0.05.

## Results

### Food intake, body weight and body composition

Obese rats fed the combination of VA+CLA showed increased food intake compared to those obese rats fed either the CD or CLA diet. Paradoxically, the VA+CLA fed rats showed reduced body weight (p < 0.001) compared to the CLA group (Table 2). Despite the higher body weight of rats fed the CLA diet as compared to obese control, no difference was observed in absolute and relative heart weights or fat pad deposition (p > 0.05), as measured by the amount of absolute and relative perirenal and inguinal fat pad weights compared with the obese rats fed the CD (Table 2). In contrast, feeding either the CLA or the VA+CLA diet resulted in a lower absolute liver weight by 15% and 26%, respectively, and both diets reduced the ratio of liver weight to total body weight by 22% as compared to obese rats fed the CD (p < 0.001).

**Table 2:** Food intake, body weight and body composition of rats in dietary groups

	Dietary groups			
	**Lean control**	**Obese control**	**Obese CLA**	**Obese VA+CLA**

Food intake (g/day)	19.7 ± 0.3^c^	32.4 ± 0.5^b^	31.4 ± 0.6^b^	36.1 ± 0.6***^a^
BW/16 wk (g)	384 ± 8.2^c^	646 ± 9.3^b^	702 ± 17.9^a^*	624 ± 9.5^b^
Heart (g)	0.89 ± 0.0^b^	1.19 ± 0.0^a^	1.26 ± 0.0^a^	1.23 ± 0.0^a^
weight, %BW	0.23 ± 0.0^a^	0.18 ± 0.0^b^	0.18 ± 0.0^b^	0.20 ± 0.0^b^
Liver (g)	9.1 ± 0.4d	23.1 ± 0.7^a^	19.7 ± 0.6***^b^	17.2 ± 0.3***^c^
weight, %BW	2.4 ± 0.1^c^	3.6 ± 0.1^a^	2.8 ± 0.1***^b^	2.8 ± 0.0***^b^
Perirenal FP^1 ^(g)	1.3 ± 0.1^c^	7.4 ± 0.4^ab^	8.6 ± 0.8^a^	6.7 ± 0.4^b^
weight, %BW	0.33 ± 0.0^b^	1.2 ± 0.1^a^	1.2 ± 0.1^a^	1.1 ± 0.1^a^
Inguinal FP (g)	1.4 ± 0.2^b^	17.9 ± 1.1^a^	18.5 ± 1.0^a^	15.7 ± 1.0^a^
weight, %BW	0.36 ± 0.0^b^	2.8 ± 0.2^a^	2.6 ± 0.1^a^	2.5 ± 0.1^a^

Values are means ± SEM, n = 8. Means in the same raw with different symbol are significantly different as compared to obese control; *P < 0.05, **P < 0.01, ***P < 0.001. Means in the same raw with different letter are significantly different among control (lean and obese) and obese treated groups (CLA and VA+CLA).

FP^1^= Fat pad

### Citrate synthase activity in liver and adipose tissue

**Figure 1:**
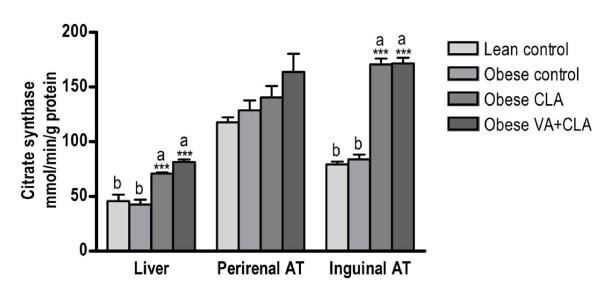
**Citrate synthase activity in liver, perirenal and inguinal adipose tissue**. Values are mean ± SEM, n = 8. Means with different symbol are significantly different as compared to obese control; *P < 0.05, **P < 0.01, ***P < 0.001. Means with different letter are significantly different among control (lean and obese) and obese treated groups (CLA and VA+CLA). AT, adipose tissue.

The activity of citrate synthase in liver and adipose tissue is shown in Figure 1. There was a higher (p < 0.001) citrate synthase activity in liver and inguinal adipose tissue after feeding either CLA or VA+CLA diets as compared to lean and obese rats fed the CD. Interestingly, the citrate synthase activity in liver and adipose tissue did not differ between lean and obese rats fed the CD (p > 0.05).

### Fatty acid profile in epididymal adipose tissue triglyceride

The fatty acid composition in adipose tissue triglyceride was shown to directly reflect the dietary fatty acid composition as shown in Table 3. Obese rats fed with the CLA and VA+CLA diets had a markedly increased proportion of the *cis*-9, *trans*-11 CLA isomer relative to the obese control group. The content of this isomer, expressed as a percentage of total fatty acids was different between the treated groups. The CLA diet showed the greatest incorporation of the cis-9,  trans-11 CLA isomer (70 fold greater than control group) compared to the  VA+CLA diet, which was only 46 fold  higher than the obese control group. Rats fed VA+CLA diet showed a greater incorporation of *trans*-11 18:1 (VA) compared to CLA and obese control rats (p < 0.001). Interestingly, rats fed the VA+CLA diet had lower proportions of linoleic acid (18:2 n6), α-linolenic acid (18:3 n3) and arachidonic acid (20:4 n6).

**Table 3:** Fatty acid composition (% of total fatty acids) of triglyceride in epididymal adipose tissue

	Dietary groups			
	**Lean control**	**Obese control**	**Obese CLA**	**Obese VA+CLA**

Fatty acid				
C18:0	13.91 ± 0.71^a^	6.36 ± 0.14^b^	5.47 ± 0.07^b^	6.31 ± 0.17^b^
C18:1 *t-*11 (VA)	0.05 ± 0.01^b^	0.02 ± 0.00^b^	0.08 ± 0.00^b^	1.72 ± 0.06***^a^
C18:1 *c-*9	28.37 ± 0.14^d^	37.9 ± 0.56^a^	32.72 ± 0.32***^c^	35.21 ± 0.49**^b^
C18:2 n6	35.76 ± 0.22^a^	20.51 ± 0.24^b^	20.15 ± 0.25^b^	15.06 ± 0.52***^c^
C18:3 n3	1.2 ± 0.03^a^	0.91 ± 0.02^b^	0.96 ± 0.02^b^	0.47 ± 0.06***^c^
CLA *c*-9, *t*-11	0.05 ± 0.01^c^	0.04 ± 0.01^c^	2.85 ± 0.04***^a^	1.91 ± 0.09***^b^
C20:4 n6	0.45 ± 0.01^a^	0.33 ± 0.25^b^	0.32 ± 0.01^b^	0.22 ± 0.0**^c^

Values are mean ± SEM, n = 8. Means in the same raw with different symbol are significantly different as compared to obese control; *P < 0.05, **P < 0.01, ***P < 0.001. Means in the same raw with different letter are significantly different among control (lean and obese) and obese treated groups (CLA and VA+CLA). Long chain n3 PUFA were not detectable.

### Fasting plasma lipid, glucose and insulin concentrations

As shown in Table 4, fasting plasma TG, TC and LDL-C were significantly lower in obese rats fed either the CLA or the VA+CLA diet, as compared to the CD. However, feeding the VA+CLA diet further reduced plasma NEFA concentration relative to the CLA fed rats and lowered TG and LDL-C concentrations not different from lean rats fed the CD (p > 0.05). The CLA or VA+CLA diet lowered fasting insulin to that comparable of lean rats (p > 0.05) and reduced total insulin concentration (AUC) after the MTT (p < 0.05). There was no statistical difference between groups for either glucose metabolism (fasting or iAUC) or the relative change (iAUC) in insulin.

**Table 4:** Fasting plasma lipid concentrations, glucose and insulin AUC after MTT and hepatic TG

	Dietary groups			
	**Lean control**	**Obese control**	**Obese CLA**	**Obese VA+CLA**

TG (mmol/L)	0.47 ± 0.0^c^	3.28 ± 0.4^a^	2.05 ± 0.3*^b^	1.4 ± 0.1***^bc^
NEFA (mmol/L)	0.20 ± 0.02^c^	0.48 ± 0.02^ab^	0.57 ± 0.03^a^	0.42 ± 0.05^b^
TC (mmol/L)	2.26 ± 0.0^c^	6.18 ± 0.4^a^	4.22 ± 0.2***^b^	4.92 ± 0.2**^b^
LDL-C (mmol/L)	0.97 ± 0.1^c^	2.28 ± 0.2^a^	1.56 ± 0.1**^b^	1.2 ± 0.1***^bc^
Fasting glucose (mmol/L)	5.94 ± 0.08	6.81 ± 0.4	5.94 ± 0.36	6.75 ± 0.72
Glucose iAUC (mmol/L.h)	45.19 ± 4.5	52.22 ± 7.8	70.25 ± 14.7	42.7 ± 33.05
Fasting insulin (μIU/L)	73.41 ± 27.4^b^	629.2 ± 165.3^a^	216 ± 13.6*^b^	372.8 ± 98.24^ab^
Insulin AUC (μIU/L.h)	8739 ± 1566 ^c^	40437 ± 4384 ^a^	24548 ± 3465*^b^	23001 ± 2744*^b^
Insulin iAUC (μIU/L.h)	4452 ± 1464	11805 ± 7603	11585 ± 3885	4451 ± 3015
Liver TG (mmol/g protein)	2.2 ± 0.3^d^	21.2 ± 1.0^a^	16.6 ± 1.1*^b^	12.1 ± 1.3***^c^

Plasma lipid and liver TG values are mean ± SEM, n = 8. Insulin AUC is mean ± SEM, n = 4. Means in the same raw with different symbol are significantly different as compared to obese control; *P < 0.05, **P < 0.01, ***P < 0.001. Means in the same raw with different letter are significantly different among control (lean and obese) and obese treated groups (CLA and VA+CLA).

### Post-prandial plasma TG response

**Figure 2:**
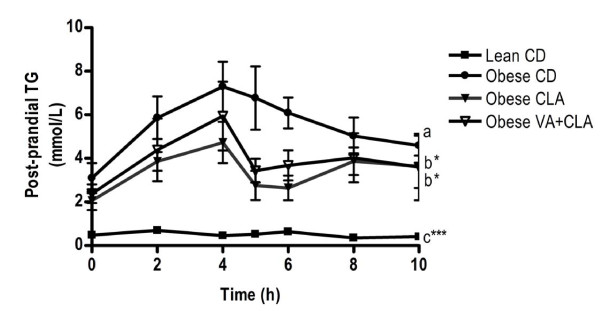
**Post-prandial triglyceride response following an oral fat challenge**. Values are mean ± SEM, n = 4. AUC differ relative to obese rats fed the CD; ^b^P < 0.05, ^c^P < 0.001. iAUC differ relative to obese rats fed the CD; ***P < 0.001.

Obese rats had a higher post-prandial plasma TG response (iAUC) compared to lean rats following an OFC (Figure 2). AUC analysis showed an improved total TG concentration (p < 0.05) over the 10-h post-prandial period in rats fed either the CLA or VA+CLA diet (35 ± 5 and 38 ± 8 mmol/L.h, respectively), compared to obese rats fed the CD (62 ± 5 mmol/L.h). However, the post-prandial iAUC for TG was not different between obese control and obese treatment groups (CLA and VA+CLA).

### Liver TG concentration and relative abundance of hepatic lipogenic enzymes

**Figure 3:**
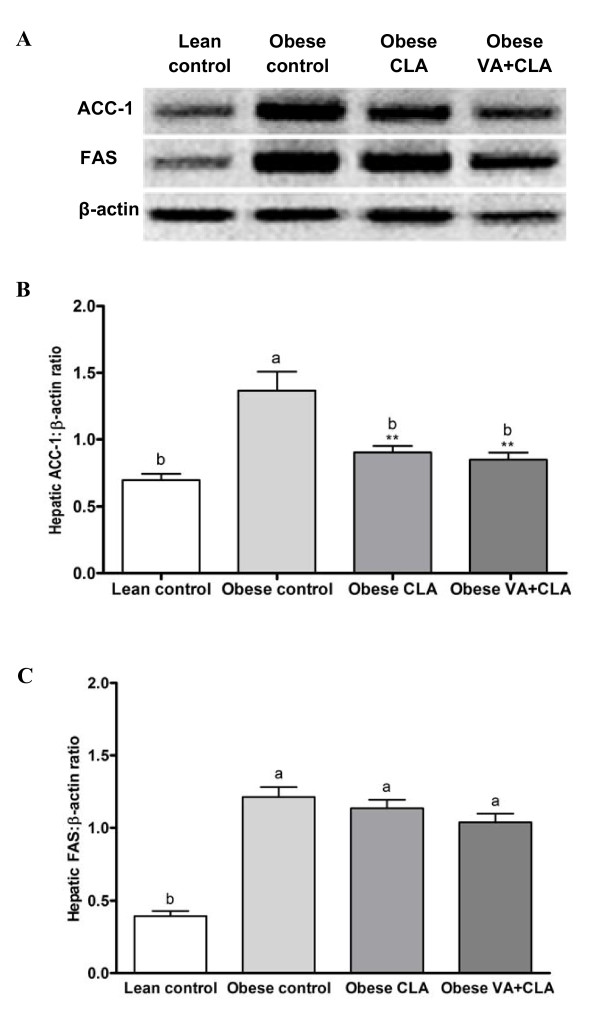
**Effects of CLA and VA+CLA on hepatic protein abundance of lipogenic enzymes**. Western blots of hepatic lipogenic enzymes (A), and relative abundance of ACC-1 (B) and FAS (C). Values are mean ± SEM, n = 8. Means with different symbol are significantly different as compared to obese rats fed the CD; *P < 0.05, **P < 0.01, ***P < 0.001. Means with different letter are significantly different among control (lean and obese) and obese treated groups (CLA and VA+CLA).

Liver TG concentration was higher in obese control rats compared to lean control rats. However, feeding the CLA diet resulted in a liver triglyceride concentration that was 22% lower than the obese rats fed the CD (Table 4). Interestingly, the VA+CLA diet further lowered liver TG concentration by 43% and 27% as compared to obese control and CLA groups, respectively. In addition, CLA and VA+CLA diets resulted in significantly lower hepatic ACC-1 protein abundance relative to obese control (34% and 38%, respectively) and was normalized to concentrations similar to lean rats fed the CD (p > 0.05) (Figure 3A, B). The relative abundance of hepatic FAS protein did not differ between the obese groups (p > 0.05) (Figure 3A, C).

## Discussion

### Combination of VA+CLA increases food intake and decreases liver weight

Dyslipidemia and insulin resistance are common features associated with cardiovascular disease and the metabolic syndrome. The homozygous obese (*cp/cp*), JCR:LA-*cp *rat, has a complete absence of the leptin receptor. As a consequence, the JCR:LA*-cp *rat spontaneously develops hyperphagia and obesity, associated dyslipidemia, insulin resistance, and macro-and micro-vascular dysfunction [34-36]. In the present study, we used this unique animal model to evaluate hypolipidemic effects of *cis*-9, *trans*-11 CLA in combination with VA. Obese rats fed the CLA diet had a higher final body weight relative to obese control rats. However, this was not associated with an increase in fat deposition. *Cis*-9, *trans*-11 CLA has been observed to regulate metabolic pathways involved in fatty acid oxidation as well as energy production and thermogenesis [37-40]; therefore we investigated whether combination VA+CLA might promote fatty acid oxidation.

### CLA and VA+CLA promote mitochondrial fatty acid oxidation in liver and adipose tissue

Peroxisome proliferator-activated receptor (PPAR)-agonists, such as thiazolidenediones (TZDs), are effective drugs for the treatment of type 2 diabetes by inducing adipogenesis as well as increasing the uptake and metabolism of free fatty acids in adipose tissue. Increased mitochondrial oxidative capacity of white adipose tissue has been observed after treatment with TZD [41,42]. CLA is a natural PPAR ligand [43-45] and has been observed to promote fatty acid oxidation [40] or TG synthesis in adipose tissue contributing to lower circulating NEFA and TG concentrations [7]. We observed a significant increase in citrate synthase activity in liver and inguinal adipose tissue following supplementation with either CLA or VA+CLA diet but not in perirenal adipose tissue. Interestingly, citrate synthase activity was not different between lean and obese control rats. It has been reported that insulin resistance is associated with an increase in muscle mitochondrial content and oxidative capacity [46-48]. Similarly, it has been demonstrated that mitochondrial biogenesis increases during adipose tissue differentiation [49]. Therefore, it can be proposed that JCR:LA*-cp *rats maintain similar mitochondrial fatty acid oxidation relative to lean rats but during increased dietary lipid consumption, it is insufficient to prevent TG deposition. Treatment with CLA or VA+CLA may stimulate mitochondrial fatty acid oxidation and in turn this may contribute to improvements in liver and adipose tissue metabolism in the JCR:LA*-cp *rat.

### Incorporation of CLA and VA in adipose tissue triglyceride

It is well established that the fatty acid composition of adipose tissue is dependent on dietary intake. However, endogenous synthesis of fatty acids, fatty acid transport and inter-conversion processes (elongation and desaturation) are also significant contributing factors to the composition of adipose tissue [50] . As expected, supplementation with *cis*-9, *trans*-11 CLA (CLA diet) and VA (VA+CLA diet) increased the proportion of these fatty acids in adipose tissue from obese rats. We also wish to note that the endogenous synthesis of *cis*-9, *trans*-11 CLA from VA may have also occurred in this study. In both humans and animals, the conversion of dietary VA to CLA has been reported to be at a rate of approximately 12-19% [12 and 14]. We also note that the dietary ratio of VA:CLA in this study was ~1.5:1, respectively (Table 1) and that the resultant incorporation of these fatty acids into adipose tissue was found to be 1:1 following supplementation (Table 3). While we cannot infer a rate of conversion from VA to CLA *per se *from this data, it would support that previously published.

### Combined dietary VA+CLA has a greater effect to reduce dyslipidemia and hepatic steatosis

One of the most striking effects of the combined treatment (VA+CLA) in the present study was the reduction in hepatic TG concentration. The improvement in fasting lipid parameters by VA+CLA diet (i.e. TG and LDL-C) suggest an additional benefit with the combination diet and is consistent with previous observations that dietary VA has lipid lowering properties independent from CLA [15]. We also wish to note that both diets (CLA and VA+CLA) consistently contained 0.15 w/w of the *trans*-10, *cis*-12 CLA isomer, which is also known for its hypolipidemic properties.

ACC-1 and FAS are two key lipogenic enzymes involved in the synthesis of fatty acids and subsequent TG synthesis. TG is then either stored as lipid droplets within the hepatocyte, secreted into the blood compartment as VLDL or hydrolyzed via oxidation [51]. It is plausible that reduced hepatic lipogenesis may contribute (at least in part) to reduced hepatic TG in rats fed either the CLA or VA+CLA diet, which is supported by a lower hepatic ACC-1 protein abundance relative to obese rats fed the CD. We have reported previously that VA may act in part via ACC-1 and FAS pathways resulting in reduced VLDL secretion to decrease circulating concentrations of plasma TG and LDL-C [15]. However, in conditions of insulin resistance, hepatic steatosis is thought to be caused by an increased free fatty acid flux from adipose tissue into the liver [52]. As discussed above, rats fed the combined treatment (VA+CLA) showed lower circulating NEFA concentrations compared to rats fed the CLA diet alone. We propose that reduced NEFA may also contribute to a further decrease in hepatic TG. In addition, activation of ACC is regulated by phosphorylation/dephosphorylation [53] and thus, it may be possible that CLA and VA+CLA diets may differently regulate post-translational modifications of ACC-1.

## Conclusion

In conclusion, results in this study confirm hypolipidemic effects of chronic supplementation with *cis*-9, *trans*-11 CLA alone or in combination with *trans*-11 VA in the dyslipidemic and insulin resistant JCR:LA*-cp *rat. Our data also support the hypothesis that a dietary formulation enriched with both CLA and VA may further enhance their hypolipidemic properties, particularly during conditions of hypertriglyceridemia, hypercholesterolemia and/or hepatic steatosis.

## List of abbreviations

ACC-1: acetyl-CoA carboxylase-1; AUC: area under the curve; iAUC: incremental area under the curve; CD: control diet; CLA: *cis*-9, *trans*-11 CLA; FAS: fatty acid synthase; MTT: meal tolerance test; NEFA: nonesterified fatty acid; OFC: oral fat challenge; TC: total cholesterol; TG: triglyceride; VA: *trans*-11 vaccenic.

## Competing interests

Funds for this work were supported in part by the Dairy Farmers of Canada, Alberta Livestock Industry Development Fund and the Natural Science and Engineering Research Council of Canada. MMJS is supported by a scholarship from the National Council of Mexico for Science and Technology (CONACyT). SDP is supported by a New Investigator Award from HSFC.

The authors declare that they have no competing interests.

## Authors' contributions

MMJS, JL, CJF, DFV, and SDP contributed to research design; MJR and JS provided essential reagents; MMJS, JL, YW, MRR and DCW conducted research and analyzed data; MMJS, CJF, DFV, and SDP contributed to the writing of the manuscript; MMJS and SDP had primary responsibility for the final content. All authors read and approved the final manuscript.

## Supplementary Material

Additional file 1 **Changes in body weight throughout the study and schematic representation of the experimental design**. Rats (n = 8) were fed a standard chow diet prior to the study (from 3-8 wk of age). Then, control and experimental diets were provided for 16 wk. An oral fat challenge test (OFC) and a meal tolerance test (MTT) were conducted on different rats (n = 4 in each test). Click here for file
