# Supplementary figures and images for: Increased hypolipidemic benefits of cis-9, trans-11 conjugated linoleic acid in combination with trans-11 vaccenic acid in a rodent model of the metabolic syndrome, the JCR:LA-cp rat
Source: Nutr Metab (Lond). 2010 Jul 16;7:60. doi: 10.1186/1743-7075-7-60 (PMC3161353; doi:10.1186/1743-7075-7-60)

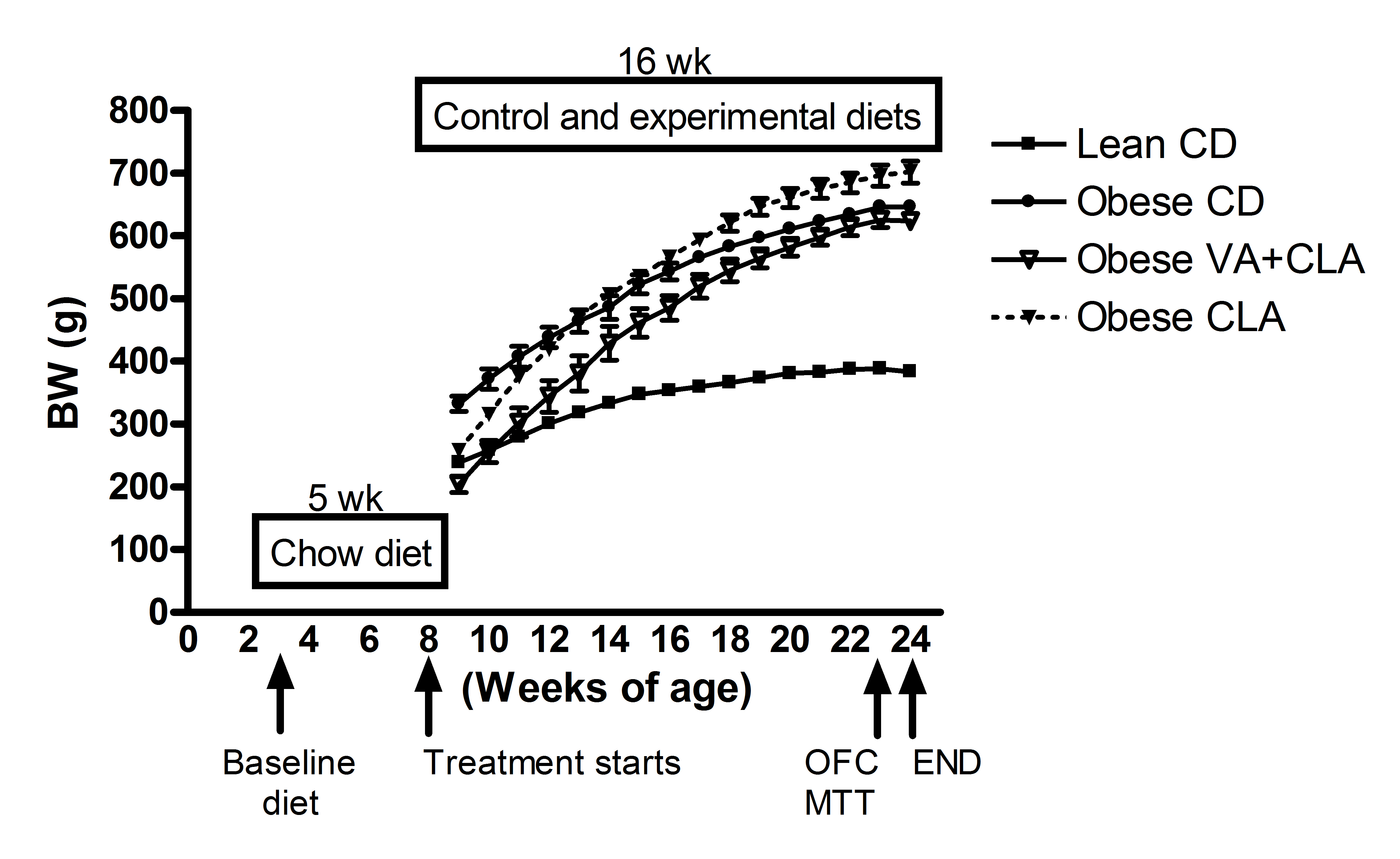

Supplement: Additional file 1 — Changes in body weight throughout the study and schematic representation of the experimental design. Rats (n = 8) were fed a standard chow diet prior to the study (from 3-8 wk of age). Then, control and experimental diets were provided for 16 wk. An oral fat challenge test (OFC) and a meal tolerance test (MTT) were conducted on different rats (n = 4 in each test). [file 1743-7075-7-60-S1.DOC]
